# Editorial: Wearable sensors for the measurement of physiological signals: what about their measurement uncertainty?

**DOI:** 10.3389/fdgth.2024.1423281

**Published:** 2024-05-08

**Authors:** Gloria Cosoli, Carlo Massaroni, Paola Saccomandi

**Affiliations:** ^1^ Department of Industrial Engineering and Mathematical Sciences, Università Politecnica delle Marche, Ancona, Italy; ^2^ Faculty of Engineering, Università Telematica eCampus, Novedrate, Italy; ^3^ Unit of Measurement and Biomedical Instrumentation, Departmental Faculty of Engineering, Università Campus Bio-Medico di Roma, Rome, Italy; ^4^ Fondazione Policlinico Universitario Campus Bio-Medico, Rome, Italy; ^5^ Department of Mechanical Engineering, Politecnico di Milano, Milan, Italy

**Keywords:** wearable sensors, health data, data analytics, metrology, accuracy, measurement uncertainty, test protocol

**Editorial on the Research Topic** Wearable sensors for the measurement of physiological signals: what about their measurement uncertainty?

This Research Topic presents a collection of studies dealing with the metrological characterization and validation of wearable sensors and devices. The application of these systems is constantly growing thanks to their multiple advantages and possibilities to be applied in many different contexts, such as sport, medicine, telemedicine, Ambient Assisted Living (AAL), comfort and well-being assessment, and personal health monitoring. The performance requirements change depending on the application, and different cost and quality segments can fit diverse targets. In any case, it is fundamental to provide the measurement results together with the related measurement accuracy to interpret them properly. What is more, the Internet of Things (IoT) capability has accelerated the spreading of wearable sensors for remote physiological monitoring and Cloud-based data processing and computing of relevant metrics, also thanks to the exploitation of Artificial Intelligence (AI) and Machine Learning (ML) algorithms.

Moreover, in the last decades many researchers have developed their own laboratory prototypes, trying to optimize specific aspects of wearable devices (e.g., decreasing susceptibility to movement artifacts). In this context, it is fundamental to be aware of the accuracy of the measurements provided by these sensors; commonly, scholars use different validation protocols, and this makes it hard to compare the results. For scientific progress in this field, it would be beneficial to define thorough validation protocols for the metrological characterization of wearables, contributing to building databases useful for multiple purposes (e.g., data mining, AI models training, analysis of uncertainty, etc.) in addition to ensure the validity and reliability of these sensors.

This Research Topic features five articles: one perspective article and four research papers. The variability of application contexts and types of sensing technologies is evident.

In their perspective article, Angelucci et al. give an overview of the digital technologies employed for step counting (e.g., pedometers and activity trackers); wearables are gaining consensus from a scientific as well as societal point of view, and the authors underline the importance of investigating their reliability in step counting used as an indicator for physical activity. In recent years, people have changed their approach towards fitness objectives by relying on wearable sensors, and this undoubtedly enhances their motivation, supporting a healthy lifestyle. This paper discusses methodological, epistemic, and ethical limitations and passes an awareness-raising message on them. The reliability and objectivity of the results should always be considered. Physical activity should be evaluated with a holistic perspective, also considering the specific target population's characteristics and factors affecting the measurement accuracy (e.g., walking speed).

On the other hand, Nassajpour et al. focus on the assessment of balance, which is essential to manage stability and coordination-related conditions and, hence, to guarantee a person's safety and proper functional status. Since traditional methods are affected by subjectivity, lack of comprehensive evaluation schemes, and remote monitoring options, the authors propose an innovative approach relying on wearable sensors (Inertial Measurement Unit—IMU—arrays) and advanced ML algorithms. High accuracy and strong correlation are found, proving that the method is reliable and effective; this can be exploited especially in environments where traditional equipment is different from being used.

Remaining in the context of gait-related measurements, Neumann et al. stress the fact that individual-specific patterns make challenging to detect gait events accurately; they tested a commercial insole system (measuring plantar pressure) in terms of accuracy, validity, and test-retest reliability performing acquisitions on chronic stroke patients and using a video camera (mounted on a rolling trolley at the height of the feet) as a ground truth for both gait events and stance duration during straight walking. The results confirm the suitability of the insole system for clinical applications, and this can be a valuable support in decision-making for stroke patients' treatments (e.g., rehabilitation pathway). This is relevant also considering that stroke is one of the leading causes of disability; moreover, the solution can be scaled to different application contexts. Transferability to real-world conditions should be investigated by considering different activities in daily life.

Indeed, if measurement accuracy is adequately taken into account, wearable sensors can also be exploited in clinical settings to support the monitoring of patients affected by diverse pathology; Unger et al. compare the performance of IMU-based sensors with optical motion capture (considered as gold standard) to evaluate a drinking task performed by stroke patients. Indeed, this movement represents a daily activity involving multiple components of the functional use of the arm. Recently, IMUs have gained a large consensus, being a low-cost and user-friendly alternative to vision-based systems. The authors find a strong agreement confirming the system applicability in clinical environments; they stress the importance of standardizing the sensors positioning for the sake of results comparison and interpretation.

It is worth noting that also a common smartphone embeds many different sensors, which can also be exploited for physiological monitoring. Hence, it can be considered a wearable device. For example, cameras can be exploited to gather photoplethysmographic (PPG) signals, from which multi-domain parameters can be inferred (e.g., heart rate and its variability, blood oxygen saturation, haemoglobin concentration, blood pressure, breathing rate, etc.). However, the accuracy of the measurements depends on multiple factors that should be investigated. Xuan et al. propose a calibration methodology for PPG performed through smartphone cameras (the finger has to cover the LED or the screen). The authors focus on tone mapping and sensor threshold; they define a calibration procedure to promote consistency and transferability among different devices. The experimental test setup comprises a bench device, including a light-blocking box and off-the-shelf LEDs. Proper calibration can significantly enhance accuracy: indeed, the authors achieve up to −74% in terms of mean absolute error on the ratio among AC and DC components, which is a fundamental property needed to accurately derive blood oxygenation and haemoglobin concentration.

These papers contribute to the research in the field of wearable devices and pay particular attention to measurement uncertainty-related aspects, which need to be continuously leveraged to provide data within a certain confidence interval.

